# Exploration of dimensionality and psychometric properties of the Pittsburgh Sleep Quality Index in cases with temporomandibular disorders

**DOI:** 10.1186/1477-7525-12-10

**Published:** 2014-01-21

**Authors:** Ksenija Rener-Sitar, Mike T John, Dipankar Bandyopadhyay, Michael J Howell, Eric L Schiffman

**Affiliations:** 1 Division of Dental Medicine, Faculty of Medicine, University of Ljubljana, Ljubljana, Slovenia; 2 University Dental Clinics, University Medical Center of Ljubljana, Ljubljana, Slovenia; 3 Division of TMD and Orofacial Pain, School of Dentistry, University of Minnesota, Minneapolis, MN, USA; 4 Division of Biostatistics, School of Public Health, University of Minnesota, Minneapolis, MN, USA; 5 Department of Neurology, University of Minnesota, Minneapolis, MN, USA

**Keywords:** Temporomandibular disorders, Sleep disorders, Factor analysis, Psychometrics, Reliability and validity, Orofacial pain, Chronic pain, Self-assessment, Questionnaires, Quality of life

## Abstract

**Background:**

This study assessed the dimensional structure of sleep quality with the Pittsburgh Sleep Quality Index (PSQI) and investigated its psychometric properties in cases with temporomandibular disorders (TMD).

**Methods:**

A convenience sample of 609 TMD cases (age: 37.1 ± 13.1 yrs, 18–67 yrs, 85% female) of the multi-center Validation Project meeting Research Diagnostic Criteria for Temporomandibular Disorders (RDC/TMD) and with sufficient PSQI data were included in this study. To investigate PSQI scores’ dimensionality, exploratory factor analysis was used. Factors were identified using the Scree plot. To investigate internal consistency, Cronbach’s alpha was calculated. Analyses were separately performed for TMD cases with (N = 496) and TMD cases withouta pain-related diagnosis (N = 113).

**Results:**

The mean PSQI score for all TMD cases was 7.1 ± 4.0 units, range: 0–19. The exploratory factor analysis identified one factor for cases with at least one pain-related TMD diagnosis as well as one factor for cases with a pain-free TMD diagnosis that explained 41% of the variance in cases with pain-related TMD and 37% in cases with pain-free TMD. Internal consistency for PSQI scores was alpha of 0.75 in cases with pain-related TMD, alpha of 0.66 in cases with pain-free TMD and alpha = 0.75 for all TMD cases.

**Conclusions:**

Sleep quality in TMD patients is a unidimensional construct and can therefore be represented by one summary score; a finding that is in line with previous reports in TMD patients.

## Background

Temporomandibular disorders (TMD) are the umbrella term encompassing mostly chronic pain conditions involving the masticatory muscles, temporomandibular joint (TMJ) an associated structures. More than half of the patients with chronic pain conditions report poor sleep quality [1]. Studies show that disruption of sleep exacerbates pain and, conversely, pain contributes to sleep disturbance [1]. Therefore, impaired sleep quality can contribute substantially to the suffering of chronic pain patients because the sleep disorders are associated with significant quality of life impairments. Patients with TMD pain are no exception. They frequently suffer from chronic orofacial pain and also have comorbid sleep disorders [2]. They commonly report poor SQ (up to 90%) [2]. Since sleep disturbances have been associated with poor treatment outcomes in the TMD patients [3], the assessment of sleep quality needs to be a part of the comprehensive evaluation of this patient population.

Subjective sleep assessment is challenging and ideally provides data on at least four characteristics of sleep: sleep initiation, sleep maintenance, sleep adequacy, and daytime somnolence. Whereas polysomnography is an objective measure of biophysiological sleep parameters, sleep quality is usually assessed using self-report. There are a number of patient-reported outcome instruments that measure various aspects of sleep quality. Seven of these instruments assess the four sleep characteristics noted above: Basic Nordic Sleep Questionnaire, Leeds Sleep Evaluation Questionnaire, Medical Outcomes Study Sleep Scale, Pittsburgh Sleep Diary, Sleep Dissatisfaction Questionnaire, Self-Rated Sleep Questionnaire, and the Pittsburgh Sleep Quality Index (PSQI) [4].

Since the introduction of the PSQI in 1989 by Buysse et al. [5] to measure sleep quality among adult psychiatric patients, this instrument has been employed in numerous other patient populations including cancer, traumatic brain injury and chronic pain patients in over 2,200 published studies. The PSQI is composed of 19 items, which are combined into seven components that are summarized into a global score that represents a unidimensional sleep quality construct [5]. However, five recent studies [6-10] have questioned the appropriateness of using only the global score. In 2006, Cole et al. examined PSQI structure by exploratory and confirmatory factor analysis in healthy and depressed elderly adults and showed initial evidence that a single global PSQI score did not capture the multidimensional nature of sleep disturbances when examined by PSQI [6]. Subsequently, four other studies explored the dimensionality of the PSQI and reported that a two- and three- factor scoring model for the PSQI were better to assess sleep quality compared to the originally proposed single-factor model (Table 1) [5].

**Table 1:** Published studies investigating two- or three- factor scoring models for the Pittsburgh Sleep Quality Index with samples description

**Study**	**Sample**	**N**	**N of factors**	**Dimensions labels**
Cole et al. [6]	USA community-dwelling depressed and nondepressed adults > 60 years	417	Three-factor	***Sleep Efficiency*** (sleep duration, habitual sleep efficiency)
				***Perceived Sleep Quality*** (subjective sleep quality, sleep latency, use of sleep medication)
				***Daily Disturbances*** (sleep disturbances, daytime dysfunction)
Aloba et al. [7]	Nigerian university students	520	Three-factor	***First factor*** (subjective sleep quality, sleep latency, habitual sleep efficiency, sleep disturbances, use of sleep medication)
				***Second factor*** (sleep duration and sleep disturbances)
				***Third factor*** (subjective sleep quality, habitual sleep efficiency, use of sleep medication)
Magee et al. [8]	Australian adults aged 18 to 59 years	364	Two- and three-factor	Same factors as Cole et al. for the three- and without *Daily Disturbances* for the two-factor model
Burkhalter et al. [9]	Swiss renal transplant recipients	135	Three-factor	Same factors as Cole et al. [6]
Mariman et al. [10]	Belgian chronic fatigue syndrome patients	413	Three-factor	Same factors as Cole et al. [6]

The individual study’s suggested PSQI dimensions labels are written in italic and bold text and the original PSQI components belonging to each suggested dimension are listed in parentheses after each dimension label.

In a sample of Nigerian university students, a three-factor model of the PSQI was identified [7], but the factors differed from Cole’s et al. study making comparisons difficult. A study assessing sleep quality in Australian adults also suggested two- and three-factor scoring models [8]. A three-factor model of the PSQI was also found to have a better fit in a sample of renal transplant recipients [9] as well as in a sample of chronic fatigue syndrome patients [10]. Cole et al. found that the PSQI factor structure has three separate factors, that is, dimensions of *sleep efficiency*, *perceived sleep quality*, and *daily disturbances* and these are reported as 3 separate scores [6].

While both two-factor and three-factor models have been reported, studies which assessed self-reported sleep disturbances of the TMD patients used a global PSQI score [2,11-14]. These findings indicate that the factor structure of this instrument in TMD patients needs to be further investigated. In particular, it is uncertain how many PSQI scores are needed to characterize sleep quality in this patient population. Moreover, the psychometric properties of the PSQI used in TMD studies are also unknown. This is important because the use of an instrument in a specific patient population is justified only if these properties are known.

The aim of this study was to assess the dimensionality and the psychometric properties of reliability and validity for the PSQI in cases with pain-related TMD and in cases with pain-free TMD.

## Methods

### Subjects

This study is a secondary data analysis, selecting from the 614 TMD cases of the multi-center RDC/TMD Validation Project [15] the 609 TMD cases with at least one RDC/TMD diagnosis [16] and a maximum of one missing PSQI question. To classify TMD cases, the RDC/TMD protocol provides criteria for classifying patients into pain-related TMD and pain-free TMD. Pain-related diagnoses include myofascial pain, temporomandibular joint (TMJ) arthralgia and TMJ osteoarthritis. Other diagnoses include osteoarthrosis and disc displacements, the latter with and without pain. The RDC/TMD is the most commonly used taxonomic classification system and use of it allows comparison with other TMD studies [15].

Cases represented a convenience sample that was recruited from both clinic and community sources (85% female, age: 37.1 ± 13.1 years). Details of the study cases and the settings have been previously reported [15]. Institutional review board's ethic approval was obtained at each of the three study sites (the University of Minnesota, the University of Washington, and the University at Buffalo) prior to initiating the RDC/TMD Validation Project [15].

The study sample of 609 TMD cases was further divided into two groups according to the presence or absence of at least one RDC/TMD pain-related diagnosis. The 496 pain-related TMD cases had at least one RDC/TMD diagnosis of myofascial pain and/or arthralgia and/or osteoarthritis. The second group of 113 TMD cases had RDC/TMD diagnosis of pain-free TMJ disc displacement and/or osteoarthrosis.

A subset of the RDC validation study cases [15] (N = 64) had retest PSQI data that were collected two weeks after the baseline examination. This interval was chosen as data from other oral health self-report instruments suggest that perceived oral health does not influence reporting substantially over short periods of repeated assessment [17,18].

### Pittsburgh Sleep Quality Index (PSQI) instrument

The PSQI is composed of 19 self-rated questions (items) and 5 questions rated by a bed partner or roommate pertaining to sleep disturbances. Only the self-rated items are used in scoring the overall scale. The self-administered scale contains 15 multiple-choice items that inquire about frequency of sleep disturbances and subjective sleep quality during the previous month. Four additional write-in items inquire about typical bedtime, wake-up time, sleep latency, and sleep duration. The 5 bed partner questions are multiple-choice ratings of sleep disturbance and are used for clinical information only. The 19 self-rated PSQI items are combined into seven components: *subjective sleep quality, sleep latency, sleep duration, habitual sleep efficiency, sleep disturbances, use of sleep medications,* and *daytime dysfunction*. Each component has a score that ranges from 0 (no difficulty) to 3 (severe difficulty). All component scores are summed to produce a global score ranging from 0–21. According to the authors of the PSQI instrument, a PSQI global score greater than 5 is suggestive of significant sleep disturbance [5]. Most patients need 5–10 minutes to complete the PSQI questionnaire. No formal training is needed to administer and score this scale [19].

### Data analysis

#### ***Item analysis of PSQI components***

An item analysis was performed according to a previously published protocol [20]. Means, standard deviations, and the proportions of the zero values were computed for each PSQI component, as well as for the global PSQI score, separately for cases with pain-related TMD and cases with pain-free TMD.

#### ***Inspection of correlations among the PSQI components***

The central characteristic of a construct is that its indicators co-vary with each other. To detect patterns among correlations indicating possible dimensions among the components scores, we inspected the polychoric correlation matrix of the PSQI component scores separately for both cases with pain-related TMD and cases with pain-free TMD [21].

#### ***Factor-analytic methods***

The PSQI components were submitted to exploratory factor analysis. Factors were extracted using the principal factors method. The Scree plot method according to Cattell [22] was used to indicate the number of factors to be extracted. If more than one factor would be extracted, factors would be rotated using the orthogonal varimax or oblique promax technique. Item loadings were examined and values larger than 0.50 were considered indicative of a relationship between the item and the associated underlying factor [23].

To assess model fit, we also performed a confirmatory factor analysis. Because data may violate the normality assumption, we used diagonally weighted least squares (DWLS) [24] and a “robust” method using the Huber-White sandwich estimator [25]. Model fit was assessed using the log-likelihood chi-square test, the standardized root mean square residual (SRMR), the root mean square error of approximation (RMSEA), the comparative fit index (CFI), and the Tucker–Lewis index (TLI). Commonly applied guidelines for adequate model fit suggest: SRMR: ≤0.08; RMSEA: ≤0.06; and CFI, TLI: ≥0.95 [26].

In addition to the assessment of dimensionality in the subgroups of cases with pain-related TMD and cases with pain-free TMD, we also performed separate analyses for women and men because gender is a major factor for TMD.

### Score reliability

#### ***Internal consistency***

Cronbach’s alpha [27] and average inter-item correlation for the Pearson correlation coefficients were computed as measures of the scores’ internal consistency. Both measures are indicators of the items’ homogeneity and indicate how strongly the items are correlated. The values of 0.7 to 0.8 are regarded as satisfactory [28] for alpha. According to Clark and Watson [29], mean inter-item correlation should fall within the range of 0.15 to 0.20 for scales that measure broad characteristics and within the range of 0.40 to 0.50 for those measuring narrower ones.

#### ***Test-retest reliability***

Temporal stability of the scores was investigated in a subset of cases from the RDC/TMD Validation Project cases with test-retest data [15]. To characterize test-retest reliability, intraclass correlation coefficients (ICC) were calculated using a one-way repeated measure ANOVA, treating the cases as a random factor. Reliability was assessed for both the instrument’s summary score and the seven PSQI components. Calculations were performed according to Shrout & Fleiss’s ICC type 2,1 [30]. Furthermore, the method of Bland and Altman [31] was used to compute the standard deviation of the differences between the first and second time points. “Limits of agreement” around the mean difference were calculated as 1.96 times the standard deviation of the differences. Hence, this statistic represents the test-retest differences expected for 95% of the individuals in the sample. If the confidence interval for the mean of the differences excluded zero, it indicated a statistically significant difference between the measures.

### Score validity

Two questions that are related to sleep from the General Health Questionnaire (GHQ) [32] were used to assess convergent validity. The questions “Have you recently lost much sleep over worry?” and “Have you recently had difficulty staying asleep?” are rated from 0 to 3 on the four-points ordinal scale, where 0 means “Not at all”, 1 – “No more than usual”, 2 – “Rather more than usual”, and 3 – “Much more than usual”. The Spearman’s rho coefficient, a nonparametric measure of statistical dependence between two quantities, was used to assess the correlation between each question and the PSQI scores. The correlation between these two items and the PSQI score was expected to be “medium” according to guidelines [33].

### Missing data and statistical software

Five of the 614 TMD cases had more than one missing PSQI item and were therefore not included in the analysis. Two hundred and thirty four cases had one missing value, which were consequently imputed using a robust median imputation within the particular PSQI item. Analyses were performed using the statistical software package STATA, (Stata Statistical Software: Release 12. College Station, TX: StataCorp LP). Any result was considered statistically significant if P < 0.05.

## Results

### Item analysis

The mean PSQI global score for all 609 TMD cases was 7.0. For the cases with pain-related TMD, the mean values for all of the seven PSQI components were higher in comparison to pain-free TMD cases (Table 2). The PSQI component that was most impaired was *sleep disturbances* with a mean of 1.5 in cases with pain-related TMD and 1.1 in cases with pain-free TMD. The least impaired PSQI component or the lowest score was reported for *sleep duration* in the cases with pain-related TMD (0.6 ± 0.9) and *use of sleep medication* in the cases with pain-free TMD (0.2 ± 0.7) indicating the latter cases used less sleep medication. Floor effects were observed frequently for all the PSQI components except for *sleep disturbances*.

**Table 2:** Descriptive statistics for the seven PSQI components and the PSQI global score (bold text) shown separately for cases with pain-related TMD and cases with pain-free TMD

**PSQI component [scale range]**	**Cases with pain-related TMD**		**Cases with pain-free TMD**	
	**(N = 496)**		**(N = 113)**	
	**Mean (SD)**	**% of 0 values**	**Mean (SD)**	**% of 0 values**
1. Subjective sleep quality [0–3]	1.2 (0.8)	17.3	0.8 (0.7)	31.9
2. Sleep latency [0–3]	1.2 (1.0)	29.0	0.8 (0.8)	45.1
3. Sleep duration [0–3]	0.6 (0.9)	58.9	0.5 (0.7)	61.1
4. Habitual sleep efficiency [0–3]	1.3 (1.3)	44.8	1.0 (1.4)	61.1
5. Sleep disturbances [0–3]	1.5 (0.6)	2.2	1.1 (0.4)	1.8
6. Use of sleep medication [0–3]	0.7 (1.1)	65.1	0.2 (0.7)	86.7
7. Daytime dysfunction [0–3]	1.0 (0.7)	23.4	0.6 (0.6)	49.6
**PSQI global score [0–21]**	**7.1 (4.0)**	**0.4**	**5.1 (3.1)**	**0.0**

### Inspection of the polychoric correlation matrix

The polychoric correlation matrix (Table 3) presented varying correlations between the seven PSQI components, ranging from -0.18 to the low 0.70 s. In cases with pain-related TMD, correlations ranged from 0.12 to 0.64. The average inter-item correlation was 0.30 in this group of cases. In cases with pain-free TMD, the correlations ranged from -0.18 to 0.74, and among them, three correlations had a negative value, although all three of them were of small magnitude. The average inter-item correlation was smaller in comparison to the cases with pain-related TMD with a value 0.22.

**Table 3:** Polychoric correlation matrices for the seven PSQI components in cases with pain-related TMD and cases with pain-free TMD are shown in the lower left triangle area, and the upper right triangle area, respectively

**PSQI component**	**1.**	**2.**	**3.**	**4.**	**5.**	**6.**	**7.**
1. Subjective sleep quality		0.54	0.55	0.26	0.74	0.06	0.54
2. Sleep latency	0.57		0.39	0.32	0.53	0.35	0.33
3. Sleep duration	0.58	0.46		0.26	0.28	0.01	0.34
4. Habitual sleep efficiency	0.33	0.36	0.51		0.26	-0.18	0.23
5. Sleep disturbances	0.64	0.47	0.45	0.17		-0.12	0.58
6. Use of sleep medication	0.32	0.36	0.30	0.12	0.39		-0.07
7. Daytime dysfunction	0.39	0.28	0.32	0.14	0.42	0.31	

### Factor-analytic methods

**Figure 1:**
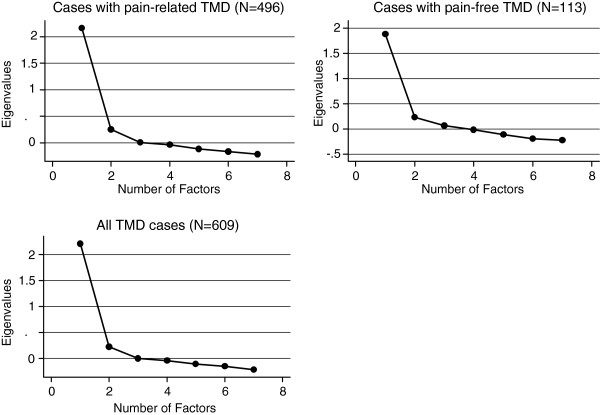
Scree plots of eigenvalues after exploratory factor analysis are shown separately for cases with pain-related TMD, cases with pain-free TMD, and for all TMD cases.

The exploratory factor analysis identified one factor for the pain-related TMD cases as well as for the pain-free TMD cases (Table 4). The factors explained 41% of the variance in the cases with pain-related TMD and 37% in the cases with pain-free TMD. Scree plots of eigenvalues plotted against the factor numbers after exploratory factor analysis are shown in Figure 1. In our confirmatory factor analysis for all TMD cases using DWLS, the unidimensional model fit the data well. The chi-square test (χ^2^ (14) = 32.746, p = 0.003) rejected the model, as we expected with our large sample size. The RMSEA was 0.05, the SRMR was 0.04, and CFI and TLI were greater than 0.95. Using the robust method for estimation, model fit worsened. The chi-square test (χ^2^ (14) = 66.235, p < 0.001) rejected the model as well, RMSEA was 0.08, SRMR was 0.04, CFI was 0.93, and TLI was 0.90. When the fit indices were compared to guideline values, all indices using DWLS estimation exceeded recommendations. For robust estimation, one index (SRMR) exceeded recommendations and four indices did not. When we performed subgroup analyses for pain-related and pain-free TMD cases as well as for women and men, model fit results showed a similar pattern in all subgroups compared with the total sample except for the pain-free TMD cases. Here, both DWLS and robust methods exceeded all guidelines values for model fit, providing strong support for the unidimensional model.

**Table 4:** One-factor model resulting from the exploratory factor analysis from the unrotated factor structure matrix for the seven PSQI components derived separately from cases with pain-related TMD (N = 496), cases with pain-free TMD (N = 113), and all TMD cases together (N = 609)

**PSQI component**	**Cases with pain-related TMD**	**Cases with pain-free TMD**	**All TMD cases**
1. Subjective sleep quality	0.83	0.88	0.84
2. Sleep latency	0.74	0.73	0.75
3. Sleep duration	0.77	0.64	0.74
4. Habitual sleep efficiency	0.52	0.47	0.51
5. Sleep disturbances	0.76	0.82	0.78
6. Use of sleep medication	0.56	0.04	0.56
7. Daytime dysfunction	0.58	0.71	0.62

### Reliability

Test-retest reliability characterized by the intraclass correlation coefficients (ICCs) was calculated for the seven PSQI components (Table 5). The median ICC for the seven PSQI components was 0.86 (range 0.57-0.91). Means of the differences between the two administrations of the questionnaire were small, ranging from -0.08 to 0.09 and were all statistically not significant. Limits of agreement indicating the interval for 95% of the test–retest differences were similar across the components and were all in the range of -1.28 to 1.36 except for the component *habitual sleep efficiency*, where the limits of agreement were larger with a range of -2.50 to 2.35.

**Table 5:** Test-retest reliability for the seven PSQI components and the PSQI global score (bold text) (N = 64 cases)

**PSQI component**	**ICC (95% CI)**	**Mean of the differences (95% CI)**	**Limits of agreement**	**p value for Pitman’s Test**
Subjective sleep quality	0.76 (0.66 to 0.86)	-0.06 (-0.19 to 0.07)	-1.12 to 1.00	0.775
Sleep latency	0.77 (0.68 to 0.87)	0.09 (-0.06 to 0.25)	-1.18 to 1.36	0.740
Sleep duration	0.73 (0.62 to 0.84)	0.03 (-0.12 to 0.18)	-1.15 to 1.21	0.214
Habitual sleep efficiency	0.57 (0.40 to 0.73)	-0.08 (-0.38 to 0.22)	-2.50 to 2.35	0.535
Sleep disturbances	0.69 (0.57 to 0.82)	0.05 (-0.07 to 0.17)	-0.92 to 1.02	0.992
Use of sleep medication	0.91 (0.87 to 0.95)	0.09 (-0.03 to 0.22)	-0.90 to 1.08	0.358
Daytime dysfunction	0.60 (0.44 to 0.75)	0.00 (-0.16 to 0.16)	-1.28 to 1.28	0.356
**PSQI global score**	**0.86 (0.80 to 0.92)**	**0.12 (-0.37 to 0.62)**	**-3.83 to 4.08**	**0.679**

Internal consistency of a PSQI was computed separately for the cases with pain-related TMD, the cases with pain-free TMD and also for the TMD cases with test-retest data. The Cronbach’s alpha value (including the one-sided confidence interval) and the average inter-item correlation were 0.75 (0.72) and 0.30 for the cases with pain-related TMD. For the cases with pain-free TMD, these values were 0.66 (0.58) and 0.22. For cases with test-retest data, values were 0.70 (0.63) and 0.25. The Cronbach’s alpha calculations with one of the seven components from the PSQI missing were also performed. When the sixth component *use of sleep medication* of the cases with pain-free TMD was omitted, the alpha value increased from 0.66 to 0.73.

### Validity

The pattern of increasing PSQI means with increasing levels of the validation questions supported PSQI summary score convergent validity (Table 6). For both global questions, similar trends were observed. As expected, the rank correlation coefficients were statistically significant and of medium magnitude.

**Table 6:** Convergent validity as assessed by correlations between two questions related to sleep from the General Health Questionnaire (GHQ) and the Pittsburgh Sleep Quality Index (PSQI) for the TMD cases sample (N = 609)

**GHQ question**	**N**	**PSQI global score(mean)**	**PSQI global score (SD)**	**Spearman’s rho (95% CI), and a level of significance**
**Have you recently lost much sleep over worry?**				0.43 (0.36 to 0.49)*
Not at all	296	5.4	3.4	
No more than usual	239	8.1	3.8	
Rather more than usual	63	9.6	4.0	
Much more than usual	11	12.5	2.7	
**Have you recently had difficulty in staying asleep?**				0.48 (0.42 to 0.54)*
Not at all	288	5.1	2.8	
No more than usual	211	7.9	3.9	
Rather more than usual	94	10.1	4.1	
Much more than usual	16	11.8	2.9	

*P < 0.001.

## Discussion

The current study demonstrated that sleep quality in TMD patients, as assessed by the PSQI, is a unidimensional scoring structure. When the PSQI data for TMD cases with or without painful diagnose were analyzed separately with exploratory factor analysis and the inspection of the scree plots, the results showed clearly a single common latent factor that explained item responses. The only exception was a low loading for the sixth component of the PSQI questionnaire called *use of sleep medication* for the cases with pain-free TMD. These individuals don’t use a lot of sleep medications, and therefore, sleep disturbances due to sleep medications are challenging to identify. Internal consistency of the PSQI was also lower in these latter cases with Cronbach’s alpha value of 0.66, which was below the threshold for the internal consistency. When the *use of sleep medication* data was omitted from the analysis, the internal consistency coefficient increased to 0.73. Sleep medication use varies across populations and may not have a strong relationship with the other variables. However, greater use of sleep medications by cases with pain-related TMD may mitigate differences in sleep quality, if the medications are effective, compared to pain-free TMD cases thus masking the true difference between these two types of cases. Conversely, cases with pain-related TMD may use more sleep medications because they use more medications in general, including pain medications, and since they see more doctors for their pain, they have more opportunity to get sleep medications.

The polychoric correlations between the *subjective sleep quality* and the *sleep disturbances*, between the s*ubjective sleep quality* and the *sleep duration*, and between the *subjective sleep quality* and the *sleep latency* had the largest magnitudes (up to 0.74). These large correlations were similar in cases with pain-related TMD and in cases with pain-free TMD.

The PSQI questionnaire comprises 19 individual items assessing sleep quality, and 15 of them are further combined into seven components. The calculation of these components is performed differently than most multi-item self-report instruments which use simple sum of their items (i.e. Oral Health Impact Profile questionnaire) [34]. A particular computation is needed for each of the seven PSQI components. This burden may potentially obscure the meaning of each component and also impede a widespread clinical application.

Our results can be generalized to other TMD populations because the RDC/TMD Validation Project data set was derived from a diverse spectrum of TMD clinic and community cases [15]. All participants were thoroughly evaluated to ensure correct TMD diagnosis necessary for inclusion as study cases [15]. Even among TMD patient populations with different distributions of TMD diagnostic subtypes, such discrepancies would not limit generalizability, because we have demonstrated that the dimensional structure of the PSQI is not substantially different between the cases with pain-related TMD and cases with pain-free TMD which is a major classification used in TMD clinical practice and research.

We found nine studies using the PSQI instrument in TMD patients [2,11,12,14,35-39]. All of these studies reported sleep quality as being one dimension represented by one global PSQI score. Some of these studies reported only a global PSQI score and defined poor sleep quality as the global PSQI score being larger than 5 [35,38,39]. However, Yatani et al. [2] used the median cutoff of a global PSQI score ≥ 10 to divide “good” and “poor sleepers”. The six remaining studies reported results for some [14,37] or for all seven PSQI subscores [11-13,36], but none of these studies reported correlations among the seven components. Thus, this is the first study to report correlations between PSQI subscores in TMD patient population. Besides reporting a global PSQI score for TMD patients, Abrahamsen et al. [37] listed also the average answers obtained from the individual questions contained in the PSQI questionnaire, e.g. Hours of sleep, Minutes before falling asleep, Number of awakenings, Number of awakenings due to pain, and Episodes of daytime sleep.

We did not find any other multiple-item instrument that has been used to assess sleep quality in the TMD patients. Although some studies have used single–item questionnaires to assess sleep quality, multi-item instruments have advantages in terms of validity and reliability compared to a single question. Therefore, there is a limit to how brief an instrument can be, and in part, depends on whether it is intended for clinical or research purposes. Although the causation is currently unknown [40], sleep quality is an important issue for successful management of TMD patients. Finally, a briefer version of the PSQI and simplification of its computation would probably popularize its clinical utility significantly.

Our study had some limitations. More than one third of our TMD patient population had one missing value in the PSQI questionnaire. The results were probably not affected substantially, because the unidimensional construct of PSQI is characterized with 15 items. For all the subjects in the analyses we had at least 14 items. It is highly unlikely that this situation may have prevented the detection of a second factor – the major alternative for a unidimensional model. We divided all the TMD diagnoses only in two categories, the pain-related TMD and pain-free TMD. The pain-related TMD diagnoses comprised a myofascial pain as well as TMJ pain, which have different clinical characteristics. When we assessed model fit for our dimensionality results not all findings agreed. All results of the exploratory factor analysis favored unidimensionality, but some indices for the confirmatory factor analysis came only close to guideline recommendations. One reason for this situation might be that our TMD cases only used sleep medication rarely and this item had the lowest correlation with the latent factor and consequently a decreased model fit. Furthermore, a convenience sampling methodology was used. Nevertheless, the psychometric properties of the PSQI were assessed in TMD patients for the first time, and dimensionality of the PSQI was graphically assessed also by the use of scree plots and two factor-analytic methods. While the majority of our methods agreed that sleep disturbances in TMD patients can be characterized with one score and we have explored dimensionality in subgroups, more sophisticated analyses of measurement invariance [41] across populations are a next step in a rigorous assessment of psychometric properties. We used a convenience sample which is inferior to a consecutive sample, but our sample size was large and our patients covered the entire spectrum of TMD patients.

In conclusion, although the PSQI instrument was initially developed for psychiatric practice and research, our study provides additional evidence that it has good psychometric properties and excellent comparability of score results with other published studies for different patient populations. For the TMD patient population, the results obtained from the PSQI questionnaire can continue to be reported in the form of one global score.

## Abbreviations

PSQI: Pittsburgh Sleep Quality Index; TMD: Temporomandibular disorders; RDC/TMD: Research Diagnostic Criteria for Temporomandibular Disorders; TMJ: Temporomandibular joint; DWLS: Diagonally weighted least squares; SRMR: Standardized root mean square residual; RMSEA: Root mean square error of approximation; CFI: Comparative fit index; TLI: Tucker–Lewis index; GHQ: General Health Questionnaire; ICC: Intraclass correlation coefficient; ANOVA: Analysis of variance.

## Competing interests

The authors declare that they have no competing interest.

## Authors’ contributions

KRS designed the study, extracted data, performed the statistical analysis, interpreted the results, and drafted the manuscript. MTJ contributed to the study design, contributed to the data analysis, interpretation of the results, and to the revision of the manuscript. DB and MJH contributed to the interpretation of the results and revised the manuscript. ELS collected data, contributed to the interpretation of the results, and revised the manuscript. All authors read and approved the final manuscript.
